# Role of the Gut Endoderm in Relaying Left-Right Patterning in Mice

**DOI:** 10.1371/journal.pbio.1001276

**Published:** 2012-03-06

**Authors:** Manuel Viotti, Lei Niu, Song-Hai Shi, Anna-Katerina Hadjantonakis

**Affiliations:** 1 Developmental Biology Program, Sloan-Kettering Institute, New York, New York, United States of America; 2 Biochemistry, Cell, and Molecular Biology Program, Weill Graduate School of Medical Sciences of Cornell University, New York, New York, United States of America; Duke University Medical Center, United States of America

## Abstract

Establishment of left-right (LR) asymmetry occurs after gastrulation commences and utilizes a conserved cascade of events. In the mouse, LR symmetry is broken at a midline structure, the node, and involves signal relay to the lateral plate, where it results in asymmetric organ morphogenesis. How information transmits from the node to the distantly situated lateral plate remains unclear. Noting that embryos lacking *Sox17* exhibit defects in both gut endoderm formation and LR patterning, we investigated a potential connection between these two processes. We observed an endoderm-specific absence of the critical gap junction component, Connexin43 (Cx43), in *Sox17* mutants. Iontophoretic dye injection experiments revealed planar gap junction coupling across the gut endoderm in wild-type but not *Sox17* mutant embryos. They also revealed uncoupling of left and right sides of the gut endoderm in an isolated domain of gap junction intercellular communication at the midline, which in principle could function as a barrier to communication between the left and right sides of the embryo. The role for gap junction communication in LR patterning was confirmed by pharmacological inhibition, which molecularly recapitulated the mutant phenotype. Collectively, our data demonstrate that Cx43-mediated communication across gap junctions within the gut endoderm serves as a mechanism for information relay between node and lateral plate in a process that is critical for the establishment of LR asymmetry in mice.

## Abstract 2

Analysis of *Sox*17 mutant mice reveals that gap junction coupling across the gut endoderm of the embryo transmits the left-right asymmetric signal from the node to the site of asymmetric organogenesis in mice.

## Author Summary

Superficially, humans, like other vertebrates, are bilaterally symmetrical. Nonetheless, the internal configuration of visceral organs reveals a stereotypical asymmetry. For example, human hearts are generally located on the left and the liver on the right side within the body cavity. How this left-right asymmetry is established is an area of interest, for both intrinsic biological significance and its medical application. In the mouse, the initial event that breaks left-right symmetry occurs at the node, a specialized organ located in the midline of the developing embryo. Somehow this initial asymmetry leads to a cascade of events that results in the activation of a genetic circuit on the left side of the embryo, which then leads to asymmetric organ formation. Here we show that the laterality information that is generated at the node is transferred to the lateral extremity of the embryo across the gut endoderm, which is the precursor tissue of the respiratory and digestive tracts and associated organs such as lungs, liver, and pancreas. *Sox17* mutant mouse embryos exhibit defects in gut endoderm formation and fail to establish left-right asymmetry. Analysis of the mutants reveals that gap junction coupling across the gut endoderm is the mechanism of left-right information relay from the midline site of symmetry breaking to the site of asymmetric organogenesis in mice.

## Introduction

The elaboration of the major axes of the embryo (anterior-posterior, dorsal-ventral, and left-right) occurs at the time of gastrulation, the morphogenetic process that forms the primary germ layers (ectoderm, mesoderm, and definitive endoderm). The molecular definition of a left-right (LR) axis precedes the establishment of an overt asymmetry in that dimension, a common feature across bilateral animals. Despite some species-specific differences, the sequence of key events and core components involved in the establishment of LR asymmetry appears conserved across vertebrates [1],[2].

An initial LR symmetry-breaking event occurs in a specialized organ located at the embryonic midline, this being the node in the mouse. Around embryonic day (E) 7.75, equivalent to the early head-fold (EHF) stage, cilia protruding from the posterior-apical surface of node cells begin rotating, thereby generating directional fluid flow within the node microenvironment [3]. Whether through interpretation of mechanical flow forces [4],[5] or asymmetric distribution of signals [3],[6], nodal flow is believed to trigger a wave of Ca^2+^ on the left side of the node [4], as well as left-biased asymmetric perinodal expression of *Nodal* [7]. By E8.5, equivalent to the ∼4 somite stage, an asymmetric readout of this symmetry-breaking event is the activation of the *Nodal/Lefty2/Pitx2* genetic cascade within the left lateral plate mesoderm (LPM) [8]. Activation of these factors is required for asymmetric organogenesis [8]. The embryonic midline is thought to act as a “midline barrier” in keeping signals confined to their respective sides, both by virtue of its morphological structure and by expressing specific factors [2].

An unresolved question in the establishment of LR asymmetry in the mouse embryo is the mechanism of communication between the midline site of symmetry-breaking and the lateral plate tissues initiating asymmetric morphogenesis. By virtue of their location, cells that lie between the node and lateral plate are likely to provide the medium for signal relay. The gut endoderm and paraxial mesoderm are attractive candidate tissues for mediating signal transmission since they lie adjacent to both the node and lateral plate mesoderm. Moreover, perinodal asymmetric events, including calcium release and *Nodal* expression, occur in endodermal cells lining the node [4],[7],[9].

Using live imaging and genetic labeling, we previously noted that the gut endoderm of the mouse embryo forms by widespread intercalation of epiblast-derived definitive endoderm (DE) cells into the overlying visceral endoderm (VE) epithelium [10]. At gastrulation, DE progenitors intercalate into the distally positioned embryonic VE epithelium, also referred to as the emVE [11],[12], which constitutes the surface cell layer of the embryo. This multifocal intercalation of DE cells leads to the widespread dispersal and dilution of emVE cells [13]. As a result, the emergent gut endoderm tissue is comprised of cells of two distinct origins: DE and emVE. Furthermore, we noted that in addition to their scattered distribution within the gut endoderm, residual emVE cells exhibited a second stereotypic distribution in that they were absent from, but congregate around, the node and midline [10],[14],[15]. Even though the precise cellular dynamics orchestrating emVE displacement at the node and midline remain unknown, these data suggest that lateral dispersal in the future gut endoderm and midline displacement in the future notochord may be regulated independently.

The *Sry*-related HMG box transcription factor Sox17 is a key conserved factor involved in endoderm formation in vertebrates [16]. Mice lacking *Sox17* have a depletion of DE cells, possess an abnormal gut tube, and die around E10.5 [17]. Interestingly, *Sox17* mutant mouse embryos also display gross morphological features commonly observed in mutants of LR asymmetry establishment, including a failure to turn from a lordotic to a fetal position, an open body wall, and cardiac defects [17]–[19]. We therefore reasoned that a detailed analysis of the *Sox17* mutant might provide further insight into the gut endoderm defects and whether gut endoderm morphogenesis and LR patterning are coupled.

Here we report that *Sox17* mutant embryos exhibit a failure in emVE dispersal as well as defects in LR patterning. We noted that widespread emVE and DE cell intercalation in the prospective gut endoderm was severely affected in mutants. By contrast, emVE displacement at the midline was not. This suggested that lateral dispersal in the future gut endoderm and midline displacement in the future notochord are likely to be distinct morphogenetic processes, the former of which requires Sox17.

One mode of communication across epithelia is through gap junctions. We identified Connexin43 (Cx43) as the predominant gap junctional constituent expressed in the endoderm at a time soon after widespread emVE and DE cell intercalation is complete, when node to LPM signal relay likely occurs. In *Sox17* mutants, we noted that Cx43 is absent in the gut endoderm. We demonstrated gap junctional coupling on both the left and right sides of the gut endoderm of wild-type embryos, but not in *Sox17* mutants. We also observed that gap junction coupling in the mesoderm was isolated from the endoderm. Since Cx43 localization within the mesoderm was comparable in wild-type and mutant embryos, we concluded that LR signals must be propagated across the endoderm epithelium. Our studies also revealed an absence of gap junctional coupling across cells at the midline in wild-type embryos, thereby providing the first functional visualization of a midline barrier in the mouse.

Collectively our observations identify the gut endoderm as a key tissue of communication between node and LPM during the establishment of LR asymmetry in the mouse. We demonstrate that Cx43-mediated gap junction coupling across the endoderm is necessary for the correct temporal and spatial propagation of asymmetric signal(s) from the node to the LPM.

## Results

### *Sox17* Mutants Fail to Establish LR Asymmetry

**Figure 1:**
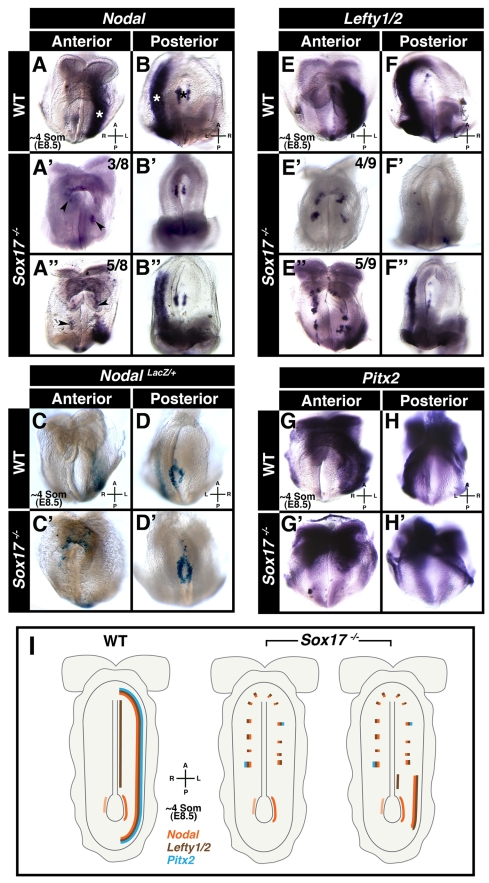
*Sox17* mutants fail to establish LR asymmetry. Panels A-B″ and E-H′ depict wholemount mRNA in situ hybridizations, and panels C-D′ depict wholemount beta-galactosidase staining. (A and B) *Nodal* in ∼4 somite stage wild-type embryo (*N* = 14), localized around node (black asterisk) and along left LPM (white asterisks). (A′–B″) *Nodal* in ∼4 somite stage Sox17 mutants, around node, and either absent (A′ and B′, *N* = 3/8) or posteriorly restricted in left LPM (A″ and B″, *N* = 5/8), and present in ectopic patches on either side of midline (black arrowheads). (C and D) *Nodal-LacZ* in left LPM and around node of wild-type embryos (*N* = 6/6). (C′ and D′) *Nodal-LacZ* around node, absent from left LPM, and in ectopic patches on either side of midline in mutants (*N* = 4/4). (E and F) *Lefty1/2* localized along left LPM and midline in wild-type embryo (*N* = 14/15). (E′–F″) *Sox17* mutants exhibited absence (E′ and F′, *N* = 4/6) or reduction (E″ and F″, *N* = 5/9) of *Lefty1/2* in left LPM. (G and H) *Pitx2* in left LPM of wild-type embryo (*N* = 7/7). (G′ and H′) In the mutant, *Pitx2* was absent from left LPM and punctate bilaterally (*N* = 5/5). (I) Diagram summarizing domains of *Nodal*, *Lefty1/2*, and *Pitx2* expression in wild-type and *Sox17* mutants. A, anterior; L, left; P, posterior; R, right.

*Sox17* mutant embryos exhibit a dysmorphic heart, have an open ventral body wall, and fail to turn (Figure S1A–S1D′) [17]–[19]. Since these features are characteristic of mutants with defects in LR patterning [8], they prompted us to determine whether LR asymmetry is established in *Sox17* mutants. To do so, we analyzed the expression of components of the core circuitry controlling the establishment of LR asymmetry in mice: genes encoding the TGFβ family proteins Nodal and Lefty2, and the homeodomain protein Pitx2. In E8.5 wild-type embryos, *Nodal* was expressed around the node and along the left LPM (Figure 1A and 1B). In stage-matched *Sox17* mutants, *Nodal* was present around the node, but absent (three out of eight embryos analyzed) or reduced and restricted posteriorly (five out of eight embryos analyzed) in the left LPM (Figure 1A′–1B″). *Sox17* mutants also exhibited ectopic patchy domains of *Nodal* expression on both the left and right sides. Notably, these ectopic patches of gene expression were not in cells of the mesoderm layer; instead, they were located superficially and were confined to the endoderm layer of the embryo, as analyzed below.

These observations were confirmed using a *Nodal^LacZ^* knock-in reporter allele [20], where lacZ activity was detected around the node and along the left LPM in *Nodal^LacZ/+^* embryos (Figure 1C and 1D). By contrast, in *Nodal^LacZ/+^;Sox17* ^−*/*−^ embryos, the reporter was present around the node but absent from the left LPM (Figure 1C′ and 1D′). Furthermore, ectopic lacZ expressing cells were observed on both sides of the midline.

Next, we analyzed expression of *Lefty1/2*, which in wild-type embryos was present in the midline and left LPM (Figure 1E and 1F). In *Sox17* mutants, *Lefty1/2* was either absent from the left LPM (four out of nine embryos analyzed) or severely reduced and truncated anteriorly and posteriorly (five out of nine embryos analyzed) (Figure 1E′–1F″). As with *Nodal*, ectopic patches of *Lefty1/2* expression were observed on both sides of the midline. *Lefty1/2* was detectable in the midline in a subset of the *Sox17* mutant embryos analyzed, but this midline domain was truncated anteriorly as compared to wild-type stage-matched embryos (Figure S1E and S1E′). In wild-type embryos *Pitx2* was present in the cephalic region and in the left LPM (Figure 1G and 1H). In *Sox17* mutants, *Pitx2* was present in the cephalic region, but was absent from the left LPM (Figure 1G′ and 1H′). Patches of ectopic *Pitx2* expression were also observed bilaterally.

Sections through mutant embryos revealed that the ectopic patches of gene expression comprised cells that were located on the embryo's surface, while in wild-type embryos gene expression was only detected in cells residing in a deeper location within the embryo, within the mesoderm (Figure S1F–S1H′). *Sox17* mutant embryos at the early somite (ESom) stage (E8.25), prior to the onset of LPM expression, already exhibited ectopic expression of *Nodal, Lefty1/2*, and *Pitx2* (Figure S1I–S1N′). Thus ectopic gene expression preceded LPM gene expression, and so could be due to a failure in the downregulation of an earlier domain of expression. Analysis of *Nodal* in early head-fold (EHF) stage (E7.75) *Sox17* mutants revealed widespread ectopic patchy expression in the gut endoderm epithelium on the surface of the embryo (Figure S1O–S1Q′). These data demonstrate that Sox17 is required for left LPM expression of the *Nodal/Lefty/Pitx2* genetic cascade operating in the establishment of LR patterning in mice, and that absence of Sox17 results in patchy ectopic expression of these genes within the gut endoderm (see summarizing cartoon in Figure 1I and Table S1).

### Aberrant Gut Endoderm Morphogenesis in *Sox17* Mutants

Having confirmed that *Sox17* mutants failed to establish LR asymmetry, we investigated whether this phenotype was linked to the endoderm defects that were previously reported [17]. To better understand the progression of gut endoderm morphogenesis in *Sox17* mutants, we used the *Afp::GFP* reporter [21]. The *Afp::GFP* transgene permits visualization of VE cells (GFP-positive), which can be discriminated from DE cells (GFP-negative) within the gut endoderm on the embryo's surface [10].

**Figure 2:**
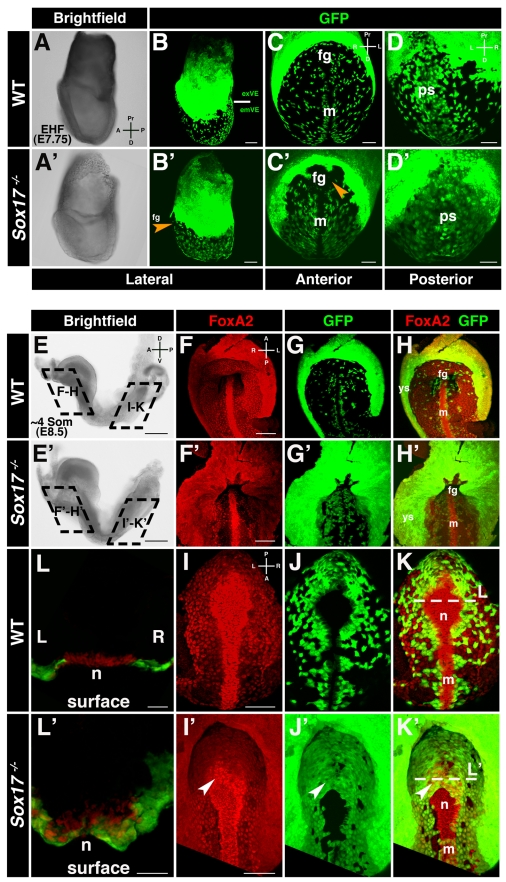
*Sox17* mutants exhibit a failure in emVE dispersal. (A and B) Brightfield image and maximum intensity projection (MIP) of confocal *z*-stacks of wild-type *Afp::GFP* (panVE-reporter) embryo at EHF. Solid white line demarcates the boundary between extraembryonic (ex) and embryonic (em) regions. (C and D) Anterior and posterior views of embryonic regions of the embryo in (B), depicting dispersed emVE cells laterally, absence of GFP at midline, and uniform distribution of GFP-positive cells overlying primitive streak. (A′–D′) Brightfield and MIPs of EHF stage *Sox17* ^−*/*−^ *; Afp::GFP^Tg/+^* embryo. A near-uniform layer of GFP-positive cells is present in extraembryonic and embryonic regions. GFP was absent in the midline and the area of the prospective anterior intestinal portal (orange arrowheads). (E and E′) ∼4 somite stage wild-type and *Sox17* mutants are comparable by gross morphology. (F–H) MIPs reveal uniform GFP in yolk sac, dispersed GFP-positive emVE cells in gut endoderm, and absence of GFP in midline of wild-type embryos. (F′–H′) In the *Sox17* mutant, while some GFP-negative areas are present, undispersed GFP-positive cells predominate in the gut endoderm around the anterior intestinal portal. Additionally, the distance from the foregut invagination to the yolk sac was decreased. (I–K) Posterior views of wild-type embryo, scattered GFP-positive emVE cells in gut endoderm, and emergent node. (I′–K′) Mutants exhibit homogeneous GFP-positive cells. Additionally, GFP-positive cells cover part of the node (white arrowheads). (L and L′) Sections through node depict FoxA2-positive cells emergent on embryo's surface in wild-type, but some remaining covered by GFP-positive cells in mutant. fg, foregut; m, midline; n, node; ps, primitive streak; ys, yolk sac; exVE, extraembryonic VE; emVE embryonic VE; A, anterior; D, distal (in panels A, C, and D); D, dorsal (in panel E); L, left; P, posterior; Pr, proximal; R, right; V, ventral. Scale bars = 200 µm in (E and E′); 100 µm in (B, B′, F, F′, I, and I′); and 50 µm in (C, C′, D, D′, L, and L′).

Since the VE encapsulates the epiblast and extraembryonic ectoderm prior to gastrulation, *Afp::GFP* embryos exhibit homogenous widespread GFP fluorescence across the entire surface of the conceptus. By the EHF stage, the cellular movements driving gut endoderm morphogenesis during gastrulation are near complete. Extraembryonic VE (exVE) GFP-positive cells positioned proximally overlying the extraembryonic ectoderm remain homogenous in the region that will form the visceral yolk sac. By contrast, GFP-positive emVE descendents located distally overlying the epiblast become dispersed and are scattered between DE cells on the embryo's surface. We analyzed EHF stage wild-type and *Sox17* heterozygous embryos that were also hemizygous for the *Afp::GFP* reporter and confirmed that the emVE had fully dispersed, as evident by a distally positioned scattered GFP-positive population of cells in the gut endoderm (Figure 2A–2D and unpublished data). By contrast in *Sox17* mutant embryos, the distally positioned emVE appeared as a uniform GFP-positive sheet on the ventral surface of the embryo (Figure 2A′–2D′). The only regions in which GFP-negative cells could be identified were around the anterior intestinal portal, the site of foregut invagination, and around the node and midline. Overall, these observations suggested that in *Sox17* mutants there was a failure to disperse the emVE within the future mid- and hindgut and that the displacement of emVE at the midline was generally unaffected.

To confirm that the *Afp::GFP* reporter was functioning as a short-term cell lineage label due to perdurance of GFP (as we noted previously, [10],[14],[15]) and exclude the possibility that GFP was being ectopically expressed by DE cells within the gut endoderm of *Sox17* mutants, we determined the localization *GFP* transcripts in *Sox17^+/+^*; *Afp::GFP^Tg/+^,* as well as *Sox17* ^−*/*−^;*Afp::GFP^Tg/+^* embryos. In head-fold (HF) stage (E7.75–8.0) wild-type *Afp::GFP* embryos, *GFP* was transcribed only in the exVE (Figure S2A). In *Sox17* mutants, *GFP* was only expressed in the exVE (Figure S2A′), indicating that no ectopic expression occurred in cells of the gut endoderm overlying the epiblast. Additionally, the pan-VE marker *Apoc2* was undetectable in the gut endoderm of both wild-type and *Sox17* mutants (Figure S2B–S2D′). These data suggest that in *Sox17* mutants, as in wild-type embryos [10], emVE cells change their state and downregulate expression of VE markers coincident with the intercalation of epiblast-derived DE. The GFP-positive cells that colonized the gut endoderm area of *Sox17* mutants were therefore emVE-derived. These cells had undergone an initial step in endoderm morphogenesis, namely cell identity reprogramming, by downregulating emVE markers, and had become poised for integration into the gut tube.

To determine if the failure to disperse the emVE persisted, we analyzed *Sox17* mutants at later embryonic stages. At the ∼4 somite stage (E8.5), wild-type embryos and *Sox17* mutants were indistinguishable by gross morphology (Figure 2E and 2E′). However, high-resolution examination of the localization of GFP-positive cells in embryos hemizygous for the *Afp::GFP* transgene revealed an aberrant distribution suggesting severe gut endoderm defects in *Sox17* mutants (Figure 2F–2L′). We simultaneously analyzed the distribution of FoxA2, a marker of gut endoderm as well as node and midline cells. Anteriorly, in wild-type embryos the region in the vicinity of the anterior intestinal portal contained sparsely scattered emVE cells (Figure 2F–2H). By contrast in *Sox17* mutants, a greater density of emVE cells was observed (Figure 2F′–2H′). Posteriorly, in the prospective hindgut region of wild-type embryos, emVE cells were dispersed, while in the midline emVE cells were displaced and congregated around the node and midline (Figure 2I–2K). By contrast, in *Sox17* mutants the epithelium on the ventral surface of the embryo was homogenously GFP-positive and so almost entirely emVE-derived. This suggested a failure in emVE cell dispersal and DE intercalation (Figure 2I′–2K′). However, the node and midline regions appeared as largely GFP-negative areas. This suggested that the morphogenetic movements of gut endoderm morphogenesis (involving emVE dispersal), and midline formation (which we propose involves emVE displacement), can be genetically uncoupled and are likely to be distinct.

Notably, closer scrutiny of the midline structures indicated that in a subset of *Sox17* mutant embryos, emergent node areas did contain some emVE-derived cells (Figure 2L–2L′). This indicates that even though gut endoderm morphogenesis and midline formation are likely to be regulated by different mechanisms, the two processes are dependent on each other for correct execution. Collectively, these observations demonstrate that defective gut endoderm morphogenesis in *Sox17* mutants occurs due to a failure to disperse the emVE. Moreover, our results reveal that the morphogenetic events that drive gut endoderm and midline morphogenesis are distinct.

### Sox17 Localizes to Gut Endoderm and Is Absent from Node and Midline

**Figure 3:**
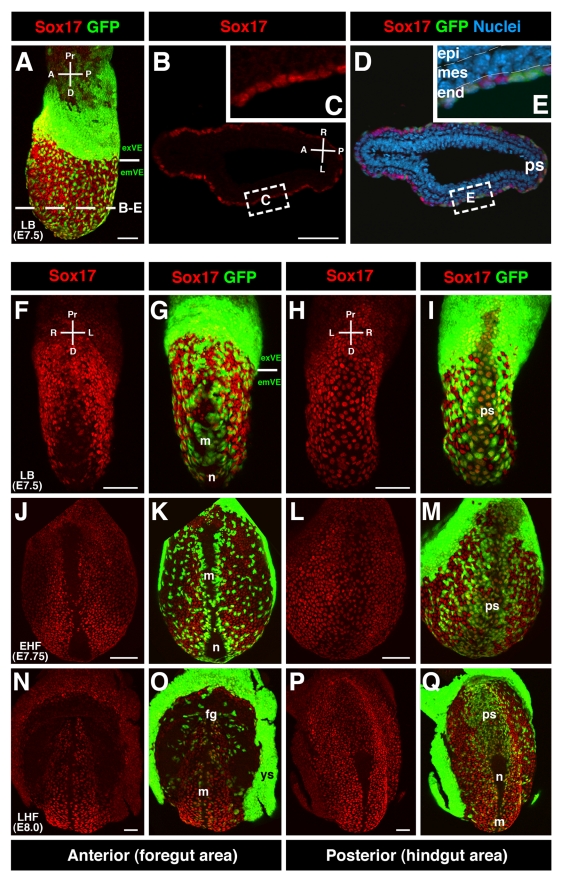
Sox17 is localized throughout gut endoderm cells, but absent from node and midline. (A) Sox17 localization specific to gut endoderm (IF, MIPs) of wild-type *Afp::GFP* embryos at LB stage. Solid line demarcates boundary between extraembryonic and embryonic regions. GFP-positive cells above are exVE, and GFP-positive cells below are emVE-derived. (B–E) Section taken at level of dashed line in (A) denotes Sox17 localization specific to endoderm, in DE cells (GFP-negative) as well as emVE cells (GFP-positive). (F and G) Anterior views of LB stage embryo showing emVE cells covering area of the prospective midline. These cells display low levels of Sox17. (H and I) Posterior views of same embryo showing Sox17-positive cells overlying the PS. (J–M) Anterior and posterior views of EHF stage embryo depicting Sox17 laterally in gut endoderm and in cells overlying the PS. Sox17 was absent from the area of the node and midline, including the vicinity of the prospective anterior intestinal portal. (N–Q) At LHF stage (E8.0) Sox17 persisted in endoderm and was absent from node and midline as well as the area of the anterior intestinal portal or the zone directly adjacent forming the boundary between extraembryonic and embryonic regions. fg, foregut; m, midline; n, node; ps, primitive streak; ys, yolk sac; epi, epiblast; mes, mesoderm; end, endoderm; exVE, extraembryonic VE; emVE, embryonic VE; A, anterior; D, distal; L, left; P, posterior; Pr, proximal; R, right. Scale bars = 100 µm.

To better understand the role of Sox17 during gut endoderm and midline morphogenesis, we determined the localization of Sox17 at the time when these tissues form. Maximum intensity projections of confocal *z*-stacks of Sox17 immunofluorescence on wild-type embryos revealed that Sox17 protein was always nuclear-localized with a high signal-to-noise ratio. At the late bud (LB) stage (E7.5), when emVE cells were dispersed within the gut endoderm of wild-type embryos, and node and midline structures were emerging and displacing emVE cells [2], Sox17 was detected in both emVE and DE cells within the gut endoderm (Figure 3A–3E, and Movie S1). Anterior views of LB stage embryos revealed the region around the midline comprised mainly of emVE cells displaying low levels of Sox17 (Figure 3F and 3G, and Movie S2). Some GFP-negative patches were present, likely corresponding to first cohorts of anterior primitive streak (APS)-derived node and midline cells having reached the embryo's surface. These cell cohorts were devoid of Sox17. Posterior views of LB stage embryos revealed uniform levels of Sox17 in all gut endoderm cells, including cells overlying the primitive streak (PS) representing the posterior visceral endoderm (PVE) domain (Figure 3H and 3I).

By the EHF stage when the node and midline had emerged (E7.75), Sox17 was present in cells of the gut endoderm, but absent from cells of both the node and the midline (Figure 3J–3M, and Movie S2). At the late head-fold (LHF) stage (E8.0), when the anterior intestinal portal begins its invagination, Sox17 was present throughout the gut endoderm except in regions of the foregut where it had become downregulated, while continuing to be undetectable in cells of the node and midline (Figure 3N–3Q). These data indicate that Sox17 localized to cells of the gut endoderm irrespective of their origin and that it was absent from cells of the node and midline. This suggested that the primary site of action of Sox17 was within the gut endoderm. The observation that Sox17 was absent from node and midline in wild-type embryos suggested that Sox17 is not involved in node morphogenesis per se. Thus, the inability of emVE cells to completely clear the node area in *Sox17* mutants was likely a secondary effect resulting from the failure of the emVE to disperse.

### Perinodal Asymmetries Are Generated in the Absence of Sox17

Since the node is the key symmetry-breaking organ, we further investigated the cells of the node and their behavior in *Sox17* mutants. This would reveal if any emVE cells failing to clear the vicinity of the node might be responsible for the LR asymmetry phenotype. We therefore determined if nodal cells contained cilia, if these cilia were motile, and if they could generate nodal flow. Furthermore we determined if asymmetric perinodal gene expression was induced around the node of *Sox17* mutants.

**Figure 4:**
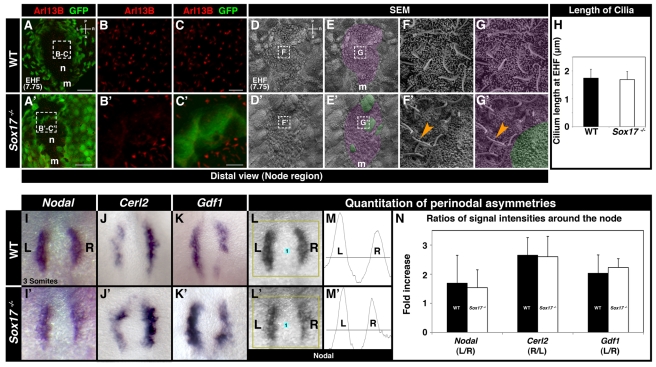
The node of *Sox17* mutants is functional. (A–C′) Localization of Arl13B in EHF (E7.75) wild-type and *Sox17* mutant embryos. (A) Distal view of the node region of wild-type embryo. (B and C) High magnification of boxed area in (A) shows Arl13B-positive puncta in the node. (A′) Distal view of *Sox17* mutant embryo showing GFP-positive cells in the node region. (B′ and C′) High magnifications of boxed area in (A′) show Arl13B-positive puncta in regions around GFP-positive cells, but no Arl13B signal within GFP-positive cells. (D–E′) SEMs of node regions of EHF wild-type and *Sox17* mutant embryos. Purple shading denotes node cells (E and E′), and green shading denotes emVE cells present overlying the node of mutants. (F and G) High magnification views of dashed boxes in (D) and (E) showing ciliated cells of the node. (F′ and G′) High magnification views of dashed boxes in (D′) and (E′) showing cilia with normal morphology (orange arrowheads) protruding from cells of the node and absence of nodal cilia on emVE cell. (H) Quantitation of nodal cilia at EHF indicating comparable lengths in wild-type (1.73±0.32 SD µm, *N* = 20) and *Sox17* mutants (1.69±0.29 SD µm, *N* = 20). (I–K′) ISH at the 3 somite stage of genes asymmetrically expressed around the node. (I–I′) Left-biased *Nodal,* (J–J′) right-biased *Cerl2*, and (K–K′) left-biased *Gdf1* expression in wild-type and mutants. (L–M′) Method for quantitation of perinodal gene expression. (L and L′) Images were imported into ImageJ (NIH) and processed with the Gel Analyzer tool. (M and M′) Graphs of *Nodal* signal intensity on left and right side of nodes. Area under the curve was used to construct histogram in (N). (N) Histogram depicting signal intensity ratios between two sides (left, L; right, R) of node. For *Nodal* and *Gdf1*, ratio was determined as L/R intensity. For *Cerl2*, ratio was determined as R/L intensity. *N* = 6 (Nodal), *N* = 3 (Gdf1, Cerl2). m, midline; n, node; A, anterior; L, left; P, posterior; R, right. Scale bars = 50 µm in (A and A′); 10 µm in (C and C′).

We analyzed the distribution of Arl13B, a small GTPase that localizes to cilia [22], in wild-type and *Sox17* mutant embryos carrying the *Afp::GFP* VE-reporter transgene at the EHF stage (E7.75), when nodes are fully formed [22]. In wild-type embryos, robust Arl13B-positive puncta were detected in cells within the node (Figure 4A–4C). We interpreted these puncta as representing the elongated cilia present on the apical surface of cells of the node. In *Sox17* mutant nodes, Arl13B-positive puncta were also present, except on emVE cells that had remained in the node region (Figure 4A′–4C′). Analysis of confocal *z*-stacks revealed an absence of robust Arl13B-positive puncta in cells that remained submerged and thus covered by emVE cells (unpublished data). Scanning electron microscopy (SEM) permitted the high-resolution visualization of nodal cilia in wild-type embryos (Figure 4D–4G). In the *Sox17* mutant, cilia with normal morphology protruded from the posterior-apical surface of node cells, except from the larger cells that resembled emVE (Figure 4D′–4G′). Quantitation revealed that the average length of nodal cilia at the EHF stage (E7.75) was comparable in wild-type and mutant embryos (Figure 4H).

Having established that cilia were present on the nodes of mutants, we investigated their motility through high-speed, high-contrast DIC imaging. At the LHF stage (E8.0), nodal cilia of wild-type embryos and *Sox17* mutants moved in a comparable clockwise motion (Movie S3). To determine if these motile cilia generated directional nodal flow, we visualized the movement of fluorescent latex beads placed on the apical surface of nodes [23]. Beads invariably migrated leftward in the nodes of both wild-type and mutant embryos (Movie S4).

Nodal flow results in left-biased perinodal gene expression asymmetries necessary to establish LR asymmetry within the LPM [7]. To determine if *Sox17* mutants generated perinodal asymmetries in gene expression, we determined the expression of *Nodal*, *Cerl2*, and *Gdf1*, three genes asymmetrically expressed around the node at the ∼2–4 somite stage. *Nodal* expression [7] was slightly higher on the left side of the node, in wild-type and *Sox17* mutants (Figure 4I and 4I′). In both wild-type and mutant embryos, *Cerl2* expression [24] was elevated on the right side of the node (Figure 4J and 4J′), while *Gdf1* expression [25] was elevated on the left side (Figure 4K and 4K′). We digitally quantified in situ hybridization signal intensities and confirmed that similar ratios of perinodal asymmetries were generated in wild-type and mutant embryos (Figure 4L–4N). These findings demonstrate that, even though in some *Sox17* mutant embryos emVE cells failed to completely clear the area of the node, the node that formed was comparable to wild-type in shape, possessed nodal cilia with normal morphology and motility, generating nodal flow, and was able to induce asymmetric perinodal gene expression.

### The Gap Junction Component Connexin43 Is Absent from Gut Endoderm of *Sox17* Mutants

Having determined that *Sox17* mutants exhibited asymmetric perinodal gene expression, we reasoned that an event downstream of the node must cause the laterality defect observed in the LPM. This implicated the gut endoderm or paraxial mesoderm, the two tissues positioned between the node and LPM, as potentially responsible for signal relay between these two distant sites. Taking into consideration that the asymmetries in the node region occurred in perinodal cells of endodermal origin [4],[7],[9], and since the Sox17 was specifically expressed by gut endoderm cells, and mutants appeared to exhibit defects specific to the gut endoderm, we favored the gut endoderm as the tissue involved. Defects within the gut endoderm might result in perturbed communication across the epithelium lying between node and lateral plate, and consequently result in a failure in the establishment of LR asymmetry.

Gap junction communication has been implicated in signal relay between the site of symmetry-breaking and tissues of asymmetric morphogenesis in several organisms, including frog, chick, and rabbit [26]. Our previous studies in the mouse demonstrated that cell-cell junctions dynamically disassemble and reassemble during emVE dispersal and concomitant DE cell intercalation takes place during gut endoderm morphogenesis [10]. We therefore investigated the presence and distribution of gap junctions within the gut endoderm of wild-type embryos and *Sox17* mutants.

Since connexins are core gap junction components, we determined which connexins were expressed in the mouse embryo around the time of LR asymmetry establishment. Expression profiling of embryonic regions of EHF stage (E7.75) wild-type embryos revealed that of the 19 characterized connexin genes, all of which were present on the array, only two were expressed above background levels (Figure S3A–S3C). The most abundant was *Gja1*, the gene encoding Connexin43 (Cx43).

**Figure 5:**
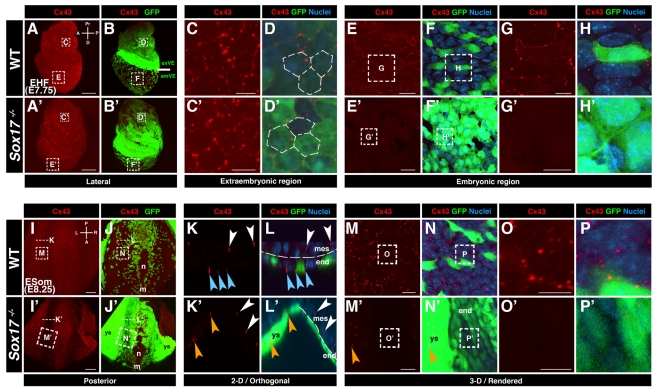
Connexin43 is not localized in the gut endoderm of *Sox17* mutants. (A, A′, B, B′) Cx43 localization in EHF stage wild-type and *Sox17* ^−*/*−^ *; Afp::GFP^Tg/+^* embryos. (C, C′, D, D′) High magnification of extraembryonic regions, depicting hexagonal distribution of Cx43-positive puncta in exVE of wild-type, as well as the *Sox17* mutant embryos. (E–H) High magnification of embryonic region of wild-type, showing Cx43 puncta at cell interfaces. (E′–H′) High magnification embryonic region of Sox17 mutant indicating absence of Cx43 puncta. (I, I′, J, J′) Cx43 localization in ESom stage (E8.25) wild-type and *Sox17* ^−*/*−^ *; Afp::GFP ^Tg/+^* embryos (posterior views). (K and L) 2-D/orthogonal views of a wild-type embryo reveal Cx43-positive puncta between endoderm (blue arrowheads) as well as mesoderm cells (white arrowheads). (K′ and L′) In the mutant, Cx43-positive puncta present between mesoderm (white arrowheads), yolk sac (orange arrowheads), but not endoderm cells. (M–P) High magnifications of wild-type gut endoderm show Cx43-positive puncta. (M′–P′) High magnifications of endoderm region in mutant reveal absence of Cx43 in endoderm, but presence in yolk sac (orange arrowheads). m, midline; n, node; ys, yolk sac; exVE, extraembryonic VE; emVE embryonic VE; A, anterior; D, distal; L, left; P, posterior; Pr, proximal; R, right. Scale bars = 100 µm in (A, A′, I, and I′); 20 µm in (E, E′, M, and M′); 10 µm in (C, C′, G, G′, K, K′, O, and O′).

We investigated the localization of Cx43 in wild-type embryos and *Sox17* mutants carrying the *Afp::GFP* VE-reporter transgene. Cx43 immunofluorescent localization was generally observed as puncta located at cell-cell interfaces. We interpret these puncta as representing gap junction complexes. Up until the onset of emVE dispersal, the distribution of Cx43 was comparable in wild-type and *Sox17* mutant embryos. Notably, Cx43 puncta were observed in all tissue layers: throughout the visceral endoderm, in the extraembryonic ectoderm, as well as within the epiblast and mesoderm (Figure S4A–S4L′ and unpublished data). By the EHF stage (E7.75), when the emVE had dispersed, Cx43 puncta were detected within exVE and gut endoderm of wild-type embryos (Figure 5A–5H). However, in *Sox17* mutants, while Cx43 puncta were detected in exVE (Figure 5A′–5D′), they were absent in the undispersed emVE (Figure 5E′–5H′). To determine if this absence of Cx43 localization persisted, we analyzed embryos at later embryonic stages. At the ESom stage (E8.25), posterior views of wild-type embryos showed Cx43 puncta in the node, midline, mesoderm, yolk sac, and all gut endoderm cells, regardless of their origin (emVE or DE) (Figure 5I–5P and Movie S5). In *Sox17* mutants, Cx43 puncta were present in the node, midline, and yolk sac, but notably were absent from the gut endoderm epithelium (Figure 5I′–5P′ and Movie S5). Collectively, these observations reveal that puncta comprising Cx43, the major gap junction component expressed in embryos of these stages, were not present amongst cells of the gut endoderm in *Sox17* mutants, suggesting a specific absence of gap junctions in this tissue.

### Gap Junction Coupling Across Gut Endoderm with a Midline Barrier

**Figure 6:**
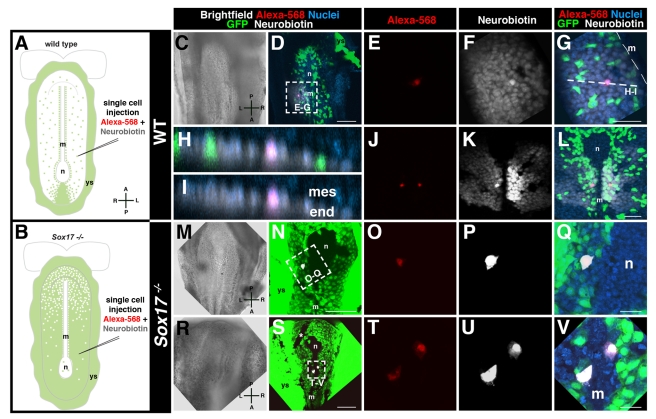
Gap junction coupling across endoderm of wild-type but not *Sox17* mutant embryos. (A and B) Experimental scheme. (C–D) Brightfield image and MIP of posterior view of ∼6 somite stage wild-type embryo, emVE cell on left side injected. (E) High magnification, injected cell retaining Alexa-568 (red). (F) Neurobiotin (white) visualized over several cell diameters. (G) Merge showing propagation of Neurobiotin through emVE-derived (GFP-positive) and the DE-derived (GFP-negative) gut endoderm cells. (H and I) 2-D/orthogonal views at dashed line in (C) showing Neurobiotin signal localized to endoderm epithelium and absent mesoderm. (J–L) Bilateral injection within gut endoderm of 0–1 somite stage wild-type embryo revealing exclusion of Neurobiotin from node/midline. (M and N) Brighfield and fluorescent merge of posterior views of a ∼5 somite stage *Sox17* ^−*/*−^ *; Afp::GFP^Tg/+^* injected into a left-sided gut endoderm cell. (O–Q) High magnifications show retention of Alexa-568 and Neurobiotin. (R–V) Bilateral injection of endoderm cells in 0–1 somite stage mutant depicting retention of Neurobiotin. GFP-negative line near the node (asterisk) is an artifact resulting from grid used for embryo positioning. m, midline; n, node; ys, yolk sac; mes, mesoderm; end, endoderm; A, anterior; L, left; P, posterior; R, right. Scale bars = 100 µm in (D, N, and S); 50 µm in (G and L); 20 µm in (Q and V).

These observations prompted us to examine gap junction communication between cells of the gut endoderm in wild-type and *Sox17* mutant embryos. To assay for gap junctional coupling, we performed single-cell iontophoretic dye injections into living embryos (Figure 6A and 6B). Two dyes, Neurobiotin and Alexa568, were co-injected into individual endoderm cells on the embryo's surface. While the high molecular weight Alexa dye remained confined within the injected cell, the lower molecular weight tracer Neurobiotin propagated intercellularly by coupling specifically through gap junctions [27],[28].

Injections into gut endoderm cells of wild-type *Afp::GFP* VE-reporter expressing embryos resulted in Neurobiotin dye propagation across several cell diameters within the gut endoderm epithelium (Figure 6C–6I). Neurobiotin propagation occurred regardless of whether an emVE or DE cell was injected. Moreover, the propagation of Neurobiotin occurred irrespective of whether cells situated on the right or left side of the embryo were injected (Figure 6J–6L). Interestingly, even though we observed Cx43 puncta in the node and midline of wild-type embryos, Neurobiotin never propagated from the gut endoderm to cells of the node and midline, and so never crossed the midline between the left to the right side of gut endoderm (Figure 6J–6L and Movie S6). The Neurobiotin signal also did not couple from the injected endoderm to adjacent mesoderm cells (Figure 6H–6I). Thus, gap junction communication within the gut endoderm was uncoupled from other germ layers and was isolated between left and right sides of the gut endoderm by a midline barrier in wild-type embryos.

We then performed dye tracer experiments on *Sox17* mutants. When individual gut endoderm cells in *Sox17* mutants were injected, they always retained both the Neurobiotin as well as the Alexa dye (Figure 6M–6Q). We consistently failed to detect dye coupling irrespective of the side of the embryo being injected (Figure 6R–6V and Movie S7). This suggested that in *Sox17* mutants, gap junction cell-cell coupling did not occur across emVE cells, likely due to the absence of Cx43 and resulting lack of functional gap junctions.

### Pharmacological Perturbation of Gap Junction Function Results in Failure to Establish LR Asymmetry

**Figure 7:**
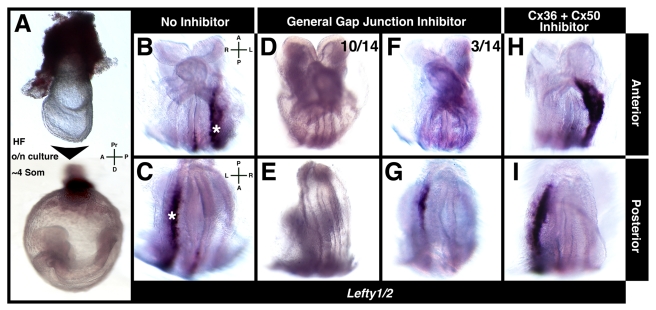
Gap junction inhibition affects LR asymmetry establishment. (A) EHF-LHF embryos cultured until the ∼4 somite stage. (B and C) *Lefty1/*2 in control embryos showing wild-type localization in left LPM (white asterisks) and midline (white arrowhead). (D–G) *Lefty1/2* expression in embryos cultured in 18 alpha-Glycyrrhetinic acid. No (D and E, 10/14) or weak/restricted signal in left LPM (F and G, 3/14). (H and I) *Lefty1/2* in embryos cultured in Mefloquine hydrochloride, exhibiting wild-type localization. A, anterior; D, distal; L, left; P, posterior; Pr, proximal; R, right.

As an independent test of whether gap junction communication was required for the establishment of LR patterning in mice, we investigated whether its pharmacological inhibition might repress signal transfer from the embryonic midline (the node) where symmetry is broken, to the lateral plate, where it is affected, and in doing so affect expression of LR asymmetry markers in the LPM. Embryos from the EHF-LHF stage (E7.75–E8.0) were cultured until the ∼4 somite stage in the presence of gap junction inhibitors (Figure 7A). To ensure that inhibitors did not interfere with node morphogenesis, we selected only embryos in which the node had emerged to the surface of the embryo. To ensure that inhibitors were acting in the temporal window during which node to lateral plate signal relay must occur and were effective before LPM gene activation, we selected only embryos in which somites had not yet formed. Two inhibitors were used: Mefloquine hydrochloride, which selectively blocks Cx36 and Cx50 [29], and 18 alpha-Glycyrrhetinic acid, a general gap junction blocker [30]. After overnight culture in the presence or absence of specific inhibitors, *Lefty1/2* expression was assayed.

Embryos cultured in the absence of inhibitors exhibited wild-type *Lefty1/2* expression (Figure 7B and 7C). By contrast, the majority of embryos cultured in 18 alpha-Glycyrrhetinic acid exhibited no *Lefty1/2* signal (10/14) (Figure 7D and 7E). Only a small subset of embryos exhibited weak *Lefty1/2* signal limited to posterior regions of the left LPM (3/14) (Figure 7F and 7G). Embryos cultured in Mefloquine hydrochloride exhibited wild-type *Lefty1/2* expression (Figure 7H and 7I). Embryos cultured in the presence of either inhibitor show normal cilia movement in the node and are able to create perinodal asymmetries (unpublished data). These observations indicated that blocking gap junction function at the time when LR asymmetry is established prevented correct LR patterning, and so molecularly recapitulated the mutant phenotype (Table S1).

## Discussion

**Figure 8:**
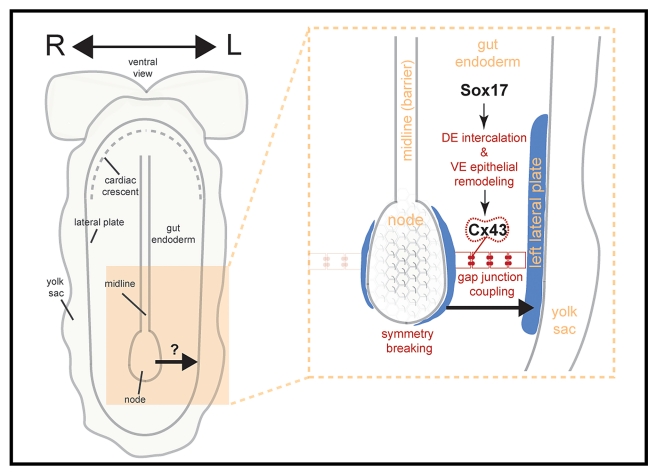
Working model for role of gut endoderm in LR asymmetry establishment. Sox17 regulates morphogenesis of the gut endoderm, whose cellular interfaces contain Cx43-comprised gap junctions. After LR symmetry is broken in the node by rotating cilia, the resulting nodal flow induces left-biased asymmetries around the node. These asymmetries are transmitted via gap junction communication within the gut endoderm to the target tissue, the left LPM, where the *Nodal/Lefty2/Pitx2* asymmetry cascade is activated.

A cascade of events establishes LR asymmetry in mice. A central unresolved question in this process is the nature of the step between symmetry-breaking at the midline and the tissues executing asymmetric morphogenesis at the lateral plate. To date, mouse mutants exhibiting LR patterning defects fall into three categories based on their expected site of gene function: the node, the LPM, and the midline [8]. No mutant affecting LR asymmetry has been reported to act within the gut endoderm, a tissue situated between the site of symmetry-breaking (the node) and the effector tissue of asymmetric morphogenesis (the lateral plate). Our studies reveal Cx43-mediated communication through gap junctions across the gut endoderm epithelium as a mechanism for information relay between node and LPM in the establishment of LR asymmetry in mice (for model, see Figure 8).

### The LR Asymmetry Phenotype of *Sox17* Mutants

Analysis of the *Nodal/Lefty/Pitx2* cascade of asymmetrically expressed genes indicated that LR asymmetry was not correctly established in *Sox17* mutants. *Pitx2*, the gene downstream in the pathway, was never detected in the left LPM of *Sox17* mutants. Interestingly, our data revealed some variability in the expression of asymmetry genes higher up in the cascade in embryos lacking Sox17. While many *Sox17* mutants exhibited no expression of *Nodal* and *Lefty1/2* in the LPM, others exhibited reduced and regionally restricted expression of these genes within the left LPM. In wild-type embryos, expression of *Nodal* in the LPM starts in a small region at the level of the node and subsequently expands anteriorly and posteriorly within the LPM [7]. The observation of reduced *Nodal* and *Lefty1/2* expression in the LPM of some *Sox17* mutant embryos revealed that the pathway had been activated, suggesting some signal was being relayed from the node to the LPM.

This raised the possibility that minimal emVE cell dispersal, observed in some mutants, may have been sufficient for gap junction communication. Dye coupling tracer experiments argued against the possibility of gap junction coupling in the *Sox17* mutant, because Neurobiotin was never observed to propagate between cells within the gut endoderm of *Sox17* mutants. Nonetheless, since it was not technically feasible to inject every cell within the gut endoderm epithelium of a single embryo, we cannot rule out the possibility that occasional gap junctional coupling may have occurred in a minor population of endoderm cells in some *Sox17* mutants. Alternatively, another connexin may partially compensate for the absence of Cx43 in *Sox17* mutants. Our expression profiling did identify *Cx31*, another connexin gene expressed at EHF stages, albeit at low levels (Figure S3). An upregulation of compensatory connexin(s) within the gut endoderm of *Sox17* mutants might permit some communication between node and LPM, allowing sufficient signal transmission to occur for minimal activation of the pathway in the left LPM. The levels of signal might not be robust enough to fully activate the cascade, preventing it from spreading within the LPM. Indeed, when wild-type embryos were cultured in the presence of inhibitors of all connexins, we observed a complete failure to activate LPM gene expression.

Our analysis of LR asymmetry markers also revealed the presence of ectopic patches of expression on both sides of the midline of *Sox17* mutants, with ectopically expressing cells situated on the embryo's surface. Notably, we never observed such ectopic expression in wild-type embryos that had been cultured in the presence of gap junction inhibitors, which we added at the HF stages, suggesting they originated from a defect preceding the emergence of the node and subsequent events of LR asymmetry establishment. In support of this, expression analysis in EHF stage *Sox17* mutants already revealed ectopic expression of *Nodal* in the endoderm. In PS stage wild-type embryos, *Nodal* is expressed in the entire emVE and then becomes downregulated [11]. This downregulation might not occur correctly in *Sox17* mutants, possibly as a consequence of the emVE not being dispersed. The ectopic *Nodal* patches are subsequently likely to induce *Lefty2* and *Pitx2*.

### Aberrant Distribution of emVE in *Sox17* Mutants During Gut Endoderm Morphogenesis

Even though some of the features have been documented [31], the precise cellular behaviors that drive node and midline morphogenesis, and in particular regarding the emergence of cells onto the embryo's surface, remain to be elucidated. It has been proposed that groups of node precursor cells emerge from the APS and insert into the emVE epithelium at the distal tip of the conceptus [31]. Once on the embryo's surface, cohorts of node cells gradually coalesce to form a single node pit [2], thereby providing a mechanism for collectively displacing emVE cells from the midline.

The absence of Sox17 from the node and midline suggested that this transcription factor is not directly involved in node and midline formation. Accordingly, the node and midline structures emerged onto the embryo's surface in *Sox17* mutants. This suggested that morphogenesis of the gut endoderm and node/midline are distinct processes and that they are uncoupled in the absence of Sox17.

We therefore interpret that failure to clear all emVE cells from the node region in a subset of mutant embryos as a secondary defect resulting from a failure in emVE dispersal. A completely emVE-derived gut endoderm might confine emVE cells to the midline, thereby hampering emergence of node progenitors onto the embryo's surface. Notably, the presence of residual emVE cells within the node of mutants did not disrupt leftward nodal flow and subsequent induction of asymmetric perinodal gene expression.

### Linking the Gut Endoderm to LR Patterning

The observation that gut endoderm of *Sox17* mutants lacked Cx43 puncta focused our attention on gap junctions as the potential cause for the failure in LR asymmetry establishment. We previously demonstrated that dynamic and widespread rearrangements of cell-cell junctions occur during the intercalation of DE cells into the overlying emVE layer during gut endoderm morphogenesis [10]. Prior to the onset of gastrulation, Cx43 puncta were detected at cell-cell interfaces within the emVE of *Sox17* mutants. Since in *Sox17* mutants the emVE did not become dispersed and would not need to be rearranged to accommodate intercalation of DE cells, we expected the Cx43 puncta to be maintained in the mutant endoderm. However, *Sox17* mutants displayed no Cx43 puncta within the endoderm between the no bud (OB) and EHF stages.

LR defects have not been described in mutants lacking *Cx43*. The original study characterizing a *Cx43* knockout mouse strain reported that mutants survive to term, dying at birth from heart defects [32]. Moreover, subsequent work revealed genetic background-related differences in the phenotypes resulting from conditional *Cx43* ablations [33]. It remains to be determined whether *Cx43* mutant animals exhibit features representing a failure of LR patterning, including *situs inversus* or heterotaxia.

Even though cells comprising the gut endoderm of *Sox17* mutants were almost exclusively of emVE origin, our findings argued that they complete an initial step in gut endoderm morphogenesis by downregulating markers of VE identity, as do emVE cells in wild-type embryos prior to their dispersal [10]. Thus in the absence of DE cells within the gut endoderm epithelium of *Sox17* mutants, a new wave of gap junction formation may have failed to occur. Further studies will be required to determine whether genes encoding gap junction components are direct targets of Sox17 or whether a failure in epithelial remodeling affects Cx43 localization. In either case, we reasoned that absence of Cx43 puncta within the gut endoderm caused a failure in gap junction coupling across this tissue in *Sox17* mutants.

### Gap Junction Coupling Across the Gut Endoderm

Our findings revealed that gap junction coupling occurs between gut endoderm cells and that this mode of communication propagates signals in both the left and right sides of the embryo. Gap junction coupling occurred across several cell diameters extending from a perinodal location to the lateral plate. Since coupling occurred between DE and emVE cells, cells of two distinct origins intercalate to form a congruent epithelium comprising the gut endoderm. Gap junction communication in the gut endoderm is planar and isolated from surrounding tissues. The observation that the gut endoderm was capable of gap junction communication on both the left and the right sides suggested that it acts as a passive medium for relay of asymmetric LR information. This is in accord with the fact that in certain mutants having defects in LR asymmetry, expression of genes in the LPM can be right-sided or bilateral [8]. If the node region, as a result of perturbed nodal flow, dictates to send asymmetry signals to the right or in both directions, the unbiased gut endoderm obediently propagates the signal to the right or to both sides. Furthermore, by revealing a lack of gap junctional coupling across the midline, our experiments also provide the first functional visualization of a midline barrier in the mouse. Importantly, the fact that some *Sox17* mutants exhibited limited expression of *Nodal* and *Lefty1/2* in the left LPM suggests that the LPM is responsive to LR patterning signals. Thus, we conclude that the LR asymmetry defect in *Sox17* mutants lies in perturbed LR signal propagation. Notably, these observations have also been noted by Saijoh and colleagues, who have gone on to demonstrate that the left LPM of *Sox17* mutants is responsive to LR patterning signals from Nodal-source cells (Y. Saijoh et al., unpublished work, personal communication).

The identity of the transmitted signal(s) is unknown: the signal that relays information must be produced in the vicinity of the node, transferred across the gut endoderm, which when reaching the interface with LPM must trigger the *Nodal/Lefty/Pitx2* cascade. One molecule shown to travel through gap junctions is calcium [34]–[36]. LR asymmetric localization of calcium has been reported in fish [37], chick [38], and mice [4],[39]. The two-cilia model of left-right asymmetry establishment in the mouse proposes that non-motile mechano-sensory perinodal cilia detect flow and elicit an asymmetric influx of Ca^2+^ ions through activity of PKD, a calcium-permeable ion channel [4],[5],[40]. Interestingly, mutants that fail to induce asymmetric calcium levels do not express LR asymmetry genes in the LPM [4],[40]. Another candidate is serotonin [41]. Serotonin has been demonstrated to cross gap junctions [42], and disruption of serotonergic signaling leads to aberrant LR patterning in frog and chick [43],[44]. Additional candidates likely exist, and further work will be required to identify the factors that travel through gap junctions across the gut endoderm to mediate LR asymmetry establishment in mice.

## Materials and Methods

### Mouse Strains

Mouse strains used were: *Sox17^cKO/cKO^* [45], *Sox2::Cre* [46], *Nodal^LacZ/+^* [20], *Afp::GFP* [21], and wild-type ICR (Taconic). The *Sox17^cKO^* strain was used to generate the null allele by crossing to the *Sox2::Cre* strain. Work on mice was subject to approval by, and carried out in accordance with guidelines from, the MSKCC IACUC.

### Embryo Recovery, Manipulation, and Culture

Embryos were dissected in DMEM-F12 (Gibco)/5% FCS (Lonza) and staged according to Downs and Davies [47]. For *ex utero* cultures EHF-LHF stage embryos were roller cultured in 50% rat serum/50% DMEM-F12 [48], a gaseous mixture of 5% O_2_; 5% CO_2_, and 90% N_2_ [49]. Inhibitors used were: 18 alpha-Glycyrrhetinic acid (1∶1,000, Sigma) and Mefloquine hydrochloride (1∶5,000, Sigma). Post-culture embryos were processed for in situ hybridization.

### In Situ Hybridization and β-Galactosidase Staining

For in situ hybridization (ISH), embryos were fixed in 4% PFA/PBS overnight at 4°C, dehydrated, and stored at −20°C. ISH was performed using antisense riboprobes [50] and standard protocols [51]. β-galactosidase staining was performed according to standard protocols [51]. For vibrating microtome sections of stained embryos, samples were placed in 30% sucrose/PBS overnight at 4°C, transferred into 0.4% gelatin/14% BSA/18% sucrose/10% glutaraldehyde, and sectioned at 16–20 µm (VT1000S, Leica).

### Immunostaining

Immunofluorescent (IF) staining was carried out as previously described in [10]. Antibodies used were: Arl13B (1∶300, gift of K. Anderson, MSKCC), Connexin43 (1∶300, Santa Cruz), FoxA2 (1∶1,000, Abcam), and Sox17 (1∶1,000, R&D Systems). Secondary Alexa-Fluor conjugated antibodies (Invitrogen/Molecular probes) were used at 1∶1,000. DNA was visualized with Hoechst 33342 (5 µg/mL, Invitrogen). For cryosectioning, fixed embryos were taken through a sucrose gradient, embedded in O.C.T. (Tissue-Tek), and sectioned at 12 µm (CM3050S, Leica).

### Image Data Acquisition, Processing, and Quantitation

Widefield images were collected with Axiocam MRc, MRm, or HSm CCD cameras (Zeiss) on a Leica MZ165FC. Laser scanning confocal images were acquired on a LSM510 META (Zeiss) as previously described [10],[15]. Fluorescence was excited with: 405 nm diode laser (Hoechst), 488 nm Argon laser (GFP), 543 nm HeNe laser (Alexa-543/555/568), and 633 nm HeNe laser (Alexa-633/647). Images were acquired using Plan-Apo 20×/NA0.75 and Fluar 5×/NA0.25 objectives. Optical sections ranged between 0.2 and 2 µm. Data were processed with AIM software (Zeiss) and assembled in Photoshop CS4 (Adobe). 3-D reconstructions of confocal *z*-stacks are depicted as maximum intensity projections (MIPs). ISH quantitations were performed with the Gel Analyzer tool (ImageJ, NIH). For scanning electron microscopy (SEM), embryos were prepared as previously described [39] and imaged with a Field Emission Supra 25 (Zeiss).

### Nodal Flow Detection

Fluorescent latex beads (0.5 µm diameter, Sigma) were placed over the node of embryos positioned ventral side up in 1% agarose submerged in culture medium. Beads were time-lapse imaged and tracked/annotated using the Manual Tracking plug-in (ImageJ, NIH). The high speed imaging system used (Zeiss HRm CCD camera, mounted on a Leica M165FC microscope and operated with Zeiss Axiovision software) did not, however, permit analysis of the relative speeds of beads being imaged.

### Expression Profiling

EHF stage embryos were bisected along the extraembryonic-embryonic junction and the embryonic portion placed in TRIzol (Invitrogen) (*N* = 3). Triplicates were hybridized to Mouse-6 Illumina arrays (Illumina Inc), and data were analyzed with Partek Genomics Suit (Partek Inc). These data are MIAME compliant and have been deposited in NCBI's Gene Expression Omnibus (GEO) [52], where it is publicly accessible through the accession number GSE33353.

### Single-Cell Iontophoretic Injections

LHF/ESom stage embryos were placed ventral side up and held in place with a metal mesh in artificial cerebral spinal fluid containing (in mM): 125 NaCl, 2.5 KCl, 1.25 KH_2_PO_4_, 1 MgCl_2_, 2 CaCl_2_, 25 NaHCO_3_, 1.3 ascorbate, 2.4 pyruvate, and 25 glucose (gassed with 95% O_2_ and 5% CO_2_) at room temperature. An Olympus BX51WI equipped with epifluorescence illumination, a CCD camera, and two water immersion objective lenses (UMPlanFI 10×/0.30W and LUMPlanFI/IR 60×/0.90W, Olympus) was used to visualize and target recording electrodes to cells. Glass recording electrodes (10–15 MΩ resistance) were filled with (in mM, pH 7.25): 130 potassium gluconate, 16 KCl, 2 MgCl_2_, 0.2 EGTA, 10 HEPES, 4 Na_2_ATP, 0.4 Na_3_GTP, 0.2% Alexa-568 (Invitrogen), and 0.5% Neurobiotin (Vector Laboratories). After obtaining a whole-cell patch recording, Alexa-568 and Neurobiotin were iontophoretically ejected through the recording electrode using anodal current repeated on (350 ms) and off (250 ms) for 420 s. Immediately after, embryos were placed in DMEM-F12 (Gibco)/5% FCS (Lonza) and live imaged.

## Supporting Information

Figure S1 Disrupted LR asymmetry in *Sox17* mutants. (A–B′) Brightfield and SEM imaging of anterior trunk regions of ∼15 somite stage (E9.0) embryos showing leftward looping of the heart tube in the wild-types and hypoplastic heart tube in *Sox17* mutants. (C–D′) Brightfield images of embryos at the ∼15 somite (E9.0) and ∼20 somite (E9.5) stage depicting wild-types in lordotic position and *Sox17* mutants with unturned configuration and open body wall. (E–H′) Images of ISH for *Lefty1/2* on wild-type and *Sox17* mutant embryos at the ∼4 somite stage (E8.5). (E) Ventral views of a wild-type showing signal in the left LPM (white asterisk) and along the midline (black arrowhead). (E′) Ventral view of a *Sox17* mutant with restricted *Lefty1/2* expression in the left LPM, showing limited *Lefty1/2* signal in the midline. (F) Section through the wild-type embryo in (E) shows *Lefty1/2* signal in the LPM. (G and H) High magnifications of the boxed region in (F) indicate absence of *Lefty1/2* signal in the endoderm layer. (F′) Section through the *Sox17* mutant embryo in (E′) shows reduced *Lefty1/2* signal in posterior regions of the left LPM and left- and right-sided blotchy signal in anterior regions. (G′ and H′) High magnifications of the boxed region in (F′) showing that *Lefty1/2* is expressed by cells on the surface of the embryo within the endoderm layer. (I–N′) ISH on wild-type and *Sox17* mutant embryos at the ESom stage (E8.25) showing ectopic, left- and right-sided patchy expression of laterality markers. (O–Q′) ISH for *Nodal* on wild-type and *Sox17* mutant embryos at the EHF stage (E7.75). (O) Ventral view of a wild-type embryo showing Nodal signal almost exclusively around the node. (P and Q) Sections through the embryo in (O) indicating the absence of *Nodal* expression in the endoderm layer. (O′) Ventral view of a *Sox17* mutant embryo showing *Nodal* signal around the node as well as blotchy signal throughout the embryonic region. (P′ and Q′) Sections through the embryo in (O′) showing several areas with *Nodal* signal in the endoderm layer. ps, primitive streak; epi, epiblast; mes, mesoderm; end, endoderm; A, anterior; D, distal; L, left; P, posterior; Pr, proximal; R, right.
(TIF)

Figure S2 Undispersed emVE cells in *Sox17* mutants downregulate VE markers. (A and A′) ISH for *GFP* at the HF stages (E7.75–8.0) in wild-type and *Sox17* mutant embryos hemizygous for the *Afp::GFP* transgene, showing stain in the extraembryonic region. (B–D′) ISH for the VE marker *Apoc2* at HF stages (E7.75–8.0) showing signal specific to the exVE in both wild-type and mutant. A, anterior; D, distal; L, left; P, posterior; Pr, proximal; R, right.
(TIF)

Figure S3 *Gja1* (Cx43) is the predominant connexin expressed at the EHF stage. (A) Brightfield image of EHF (E7.75) embryo depicting region used for expression profiling. (B) Tabulated normalized detection levels of genes encoding connexins in expression array (*p*-value <0.01). (C) Graphical representation of connexins gene expression levels indicating high expression of *Gja1* (Cx43) and low expression of *Gjb3* (Cx31). Expression levels for all other connexin genes fell below background cut-off (dashed line, C = 6.81). A, anterior; D, distal; P, posterior; Pr, proximal. Scale bar = 100 µm.
(TIF)

Figure S4 Cx43 localization in Sox17 mutants is normal until the onset of endoderm morphogenesis. (A and B) MIPs of confocal *z*-stacks of PS stage (6.25) wild-type and *Sox17* mutant embryos immunofluorescently stained for the gap junction component Cx43. (C, C′, D, D′) High magnifications of the emVE region showing Cx43-positive puncta in the wild-type as well as the *Sox17* mutant. (E, E′, F, F′) 2-D/orthogonal views of dashed lines in (C–D′) showing Cx43-positive puncta in the mesoderm and endoderm layers of the wild-type as well as the *Sox17* mutant. (G and H) Lateral views of OB stage (E7.25) wild-type embryos stained for Cx43, showing first stages of emVE dispersal. (I and J) High magnifications of the dashed boxes in (G) and (H) showing Cx43-positive puncta in the endoderm layer, which is in the process of being dispersed. (K and L) 2-D/orthogonal views of the dashed lines in (I) and (J) showing Cx43-positive puncta in the mesoderm layer as well as between cells of the endoderm layer. (G′ and H′) Lateral views of an OB stage (E7.25) *Sox17* mutant stained for Cx43. (I′ and J′) High magnifications of the dashed boxes in (G′) and (H′), showing CX43-positive puncta amongst undispersed emVE cells. (K′ and L′) 2-D/orthogonal views through the dashed line in (I′) and (J′) showing Cx43-positive puncta in the mesoderm layer as well as between undispersed emVE cells. emVE, embryonic VE; A, anterior; D, distal; P, posterior; Pr, proximal. Scale bars = 50 µm in (A, A′, G, and G′); 20 µm in (C, C′, I, and I′).
(TIF)

Movie S1 Sox17 localizes to gut endoderm cells. Left panel shows MIP of confocal *z*-stacks of a LB stage (E7.5) *Afp::GFP* wild-type embryo immunofluorescently stained for Sox17. All gut endoderm cells, whether emVE (GFP-positive) or DE cells (GFP-negative), show Sox17 localization. Right panel shows section taken through dashed line, showing Sox17 signal specifically in the endoderm layer. ps, primitive streak; D, distal; L, left; Pr, proximal; R, right.
(MOV)

Movie S2 Absence of Sox17 from node and midline. MIPs of anterior views of *Afp::GFP* wild-type embryos immunofluorescently stained for Sox17. Left panel shows LB stage (E7.5) embryo, displaying GFP-positive (emVE) cells overlying the midline exhibiting low levels of Sox17. Right panel showing EHF stage (E7.75) embryo, displaying a node and midline area devoid of Sox17 signal as well as clear of GFP-positive (emVE) cells. m, midline; D, distal; L, left; Pr, proximal; R, right.
(MOV)

Movie S3 The nodes of *Sox17* mutants contain beating cilia. Time-lapse of node regions of LHF stage (E8.0) embryos in culture to visualize cilia. The two upper panels display several beating cilia in nodes of wild-type (left) and *Sox17* mutant (right) embryos. The lower panels show high magnifications of a single wild-type (left) and *Sox17* mutant (right) cilium beating in a clockwise direction. For each individual panel, anterior is on bottom, left is on the left side, posterior is on the top, and right is on the right side.
(MOV)

Movie S4 *Sox17* mutant nodes generate nodal flow. Time-lapse of node regions of ESom stage (E8.25) embryos in culture. Fluorescent latex beads were dropped on the node. In the wild-type (left panel) as well as in the *Sox17* mutant (right panel), the beads migrate unidirectionally to the left side of the nodes. In the second sequence, bead movement was analyzed using tracking software (ImageJ), and the trajectories of beads are displayed. A, anterior; L, left; P, posterior; R, right.
(MOV)

Movie S5 The gap junction component Cx43 is absent from the Sox17 mutant gut endoderm. MIPs of posterior views of ESom stage (E8.25) *Afp::GFP* embryos immunofluorescently stained for Cx43. The left panel shows a wild-type embryo, displaying Cx43-positive puncta in the gut endoderm, as well as in the node, midline, and yolk sac. The right panel shows a *Sox17* mutant, displaying Cx43-positive puncta in the node, midline, and yolk sac. The gut endoderm is devoid of Cx43 puncta, except the area directly posterior to the node. A, anterior; L, left; P, posterior; R, right.
(MOV)

Movie S6 The node and midline are barriers to gap junction communication in the gut endoderm. MIPs of posterior views of an ESom stage (E8.25) *Afp::GFP* wild-type embryo. A combination of Alexa-568 and Neurobiotin was iontophoretically injected into a GFP-negative (DE lineage) cell in the left side of the gut endoderm. The left panel shows a static image of the posterior area, and the right panel shows a magnification of the boxed region. Note cellular retention of the Alexa-568 molecule and intercellular propagation of Neurobiotin. The Neurobiotin signal stops abruptly at the periphery of the node and the midline. m, midline; A, anterior; L, left; P, posterior; R, right.
(MOV)

Movie S7 Gap junction communication in the gut endoderm is blocked in *Sox17* mutants. The two sequences correspond to Figure 6G and 6V. Left panel depicting the wild-type, showing spread of Neurobiotin over several cell diameters within the gut endoderm layer. The GFP signal in the injected emVE cell is low due to leakage of the GFP protein out of the cell during the injection process. Right panel depicting the *Sox17* mutant, showing retention of Neurobiotin in the injected cells. A, anterior; L, left; P, posterior; R, right.
(MOV)

Table S1 Tabulation of experimental results.
(DOC)

## Abbreviations

APS: anterior primitive streak
Cx43: Connexin43
DE: definitive endoderm
EHF: early head-fold
HF: head-fold
IF: Immunofluorescent
ISH: in situ hybridization
LB: late bud
LHF: late head-fold
LPM: lateral plate mesoderm
LR: left-right
MIPs: maximum intensity projections
PS: primitive streak
PVE: posterior visceral endoderm
SEM: scanning electron microscopy
VE: visceral endoderm
