# Supplementary figures and images for: Role of the Gut Endoderm in Relaying Left-Right Patterning in Mice
Source: PLoS Biol. 2012 Mar 6;10(3):e1001276. doi: 10.1371/journal.pbio.1001276 (PMC3295824; doi:10.1371/journal.pbio.1001276)

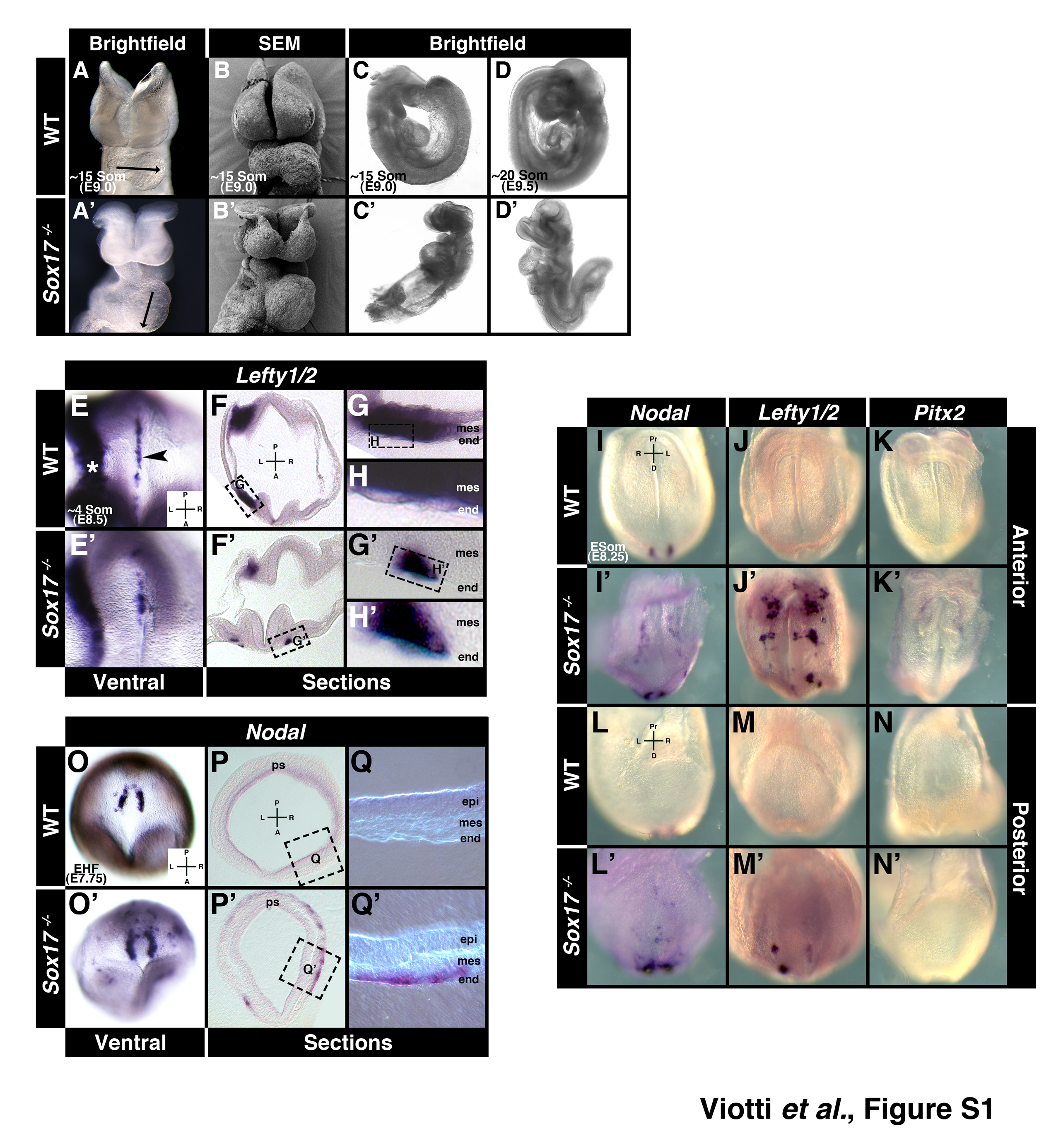

Supplement: Figure S1 — Disrupted LR asymmetry in Sox17 mutants. (A–B′) Brightfield and SEM imaging of anterior trunk regions of ∼15 somite stage (E9.0) embryos showing leftward looping of the heart tube in the wild-types and hypoplastic heart tube in Sox17 mutants. (C–D′) Brightfield images of embryos at the ∼15 somite (E9.0) and ∼20 somite (E9.5) stage depicting wild-types in lordotic position and Sox17 mutants with unturned configuration and open body wall. (E–H′) Images of ISH for Lefty1/2 on wild-type and Sox17 mutant embryos at the ∼4 somite stage (E8.5). (E) Ventral views of a wild-type showing signal in the left LPM (white asterisk) and along the midline (black arrowhead). (E′) Ventral view of a Sox17 mutant with restricted Lefty1/2 expression in the left LPM, showing limited Lefty1/2 signal in the midline. (F) Section through the wild-type embryo in (E) shows Lefty1/2 signal in the LPM. (G and H) High magnifications of the boxed region in (F) indicate absence of Lefty1/2 signal in the endoderm layer. (F′) Section through the Sox17 mutant embryo in (E′) shows reduced Lefty1/2 signal in posterior regions of the left LPM and left- and right-sided blotchy signal in anterior regions. (G′ and H′) High magnifications of the boxed region in (F′) showing that Lefty1/2 is expressed by cells on the surface of the embryo within the endoderm layer. (I–N′) ISH on wild-type and Sox17 mutant embryos at the ESom stage (E8.25) showing ectopic, left- and right-sided patchy expression of laterality markers. (O–Q′) ISH for Nodal on wild-type and Sox17 mutant embryos at the EHF stage (E7.75). (O) Ventral view of a wild-type embryo showing Nodal signal almost exclusively around the node. (P and Q) Sections through the embryo in (O) indicating the absence of Nodal expression in the endoderm layer. (O′) Ventral view of a Sox17 mutant embryo showing Nodal signal around the node as well as blotchy signal throughout the embryonic region. (P′ and Q′) Sections through the embryo in (O′) showing several areas w [file pbio.1001276.s001.tif]

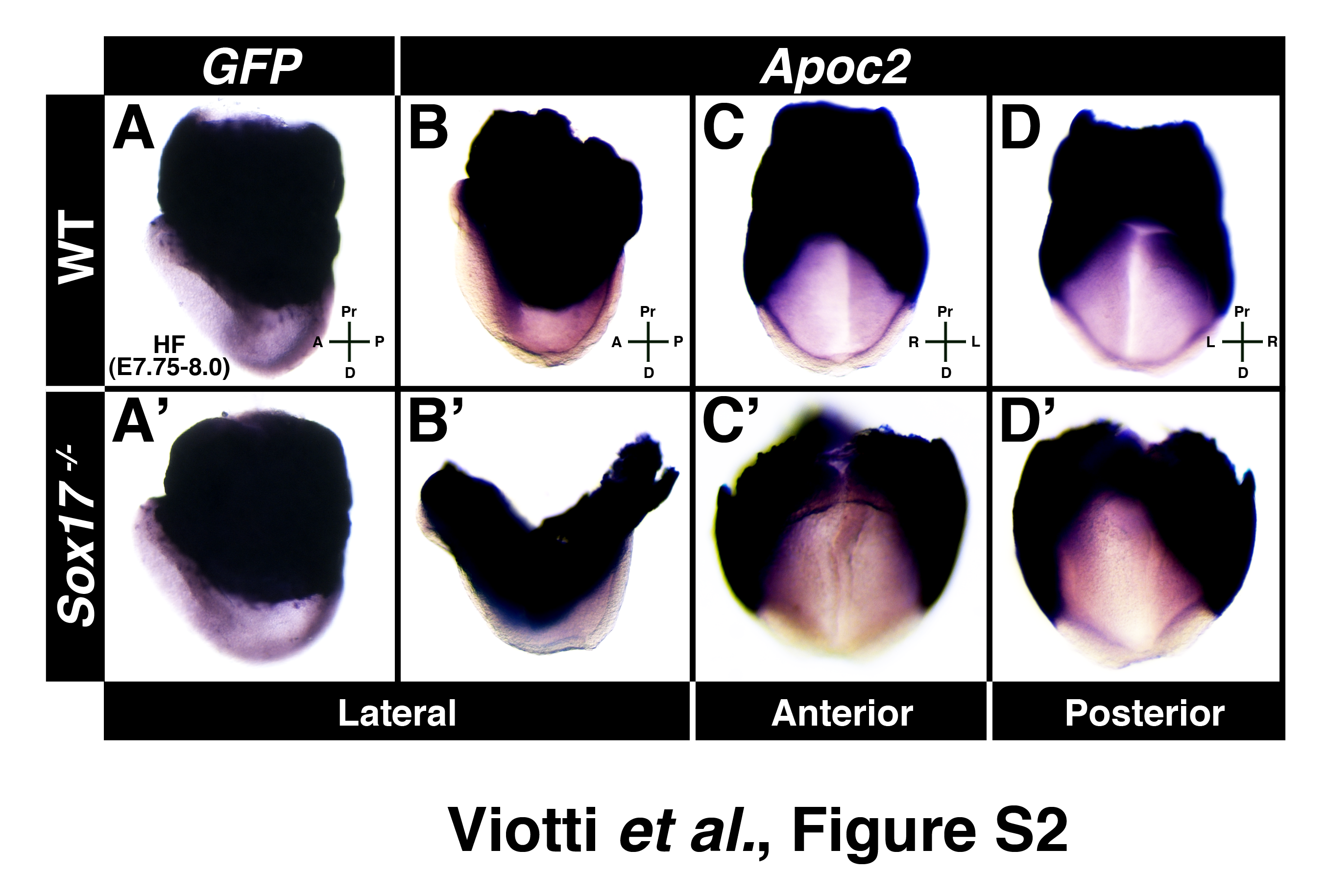

Supplement: Figure S2 — Undispersed emVE cells in Sox17 mutants downregulate VE markers. (A and A′) ISH for GFP at the HF stages (E7.75–8.0) in wild-type and Sox17 mutant embryos hemizygous for the Afp::GFP transgene, showing stain in the extraembryonic region. (B–D′) ISH for the VE marker Apoc2 at HF stages (E7.75–8.0) showing signal specific to the exVE in both wild-type and mutant. A, anterior; D, distal; L, left; P, posterior; Pr, proximal; R, right. (TIF) [file pbio.1001276.s002.tif]

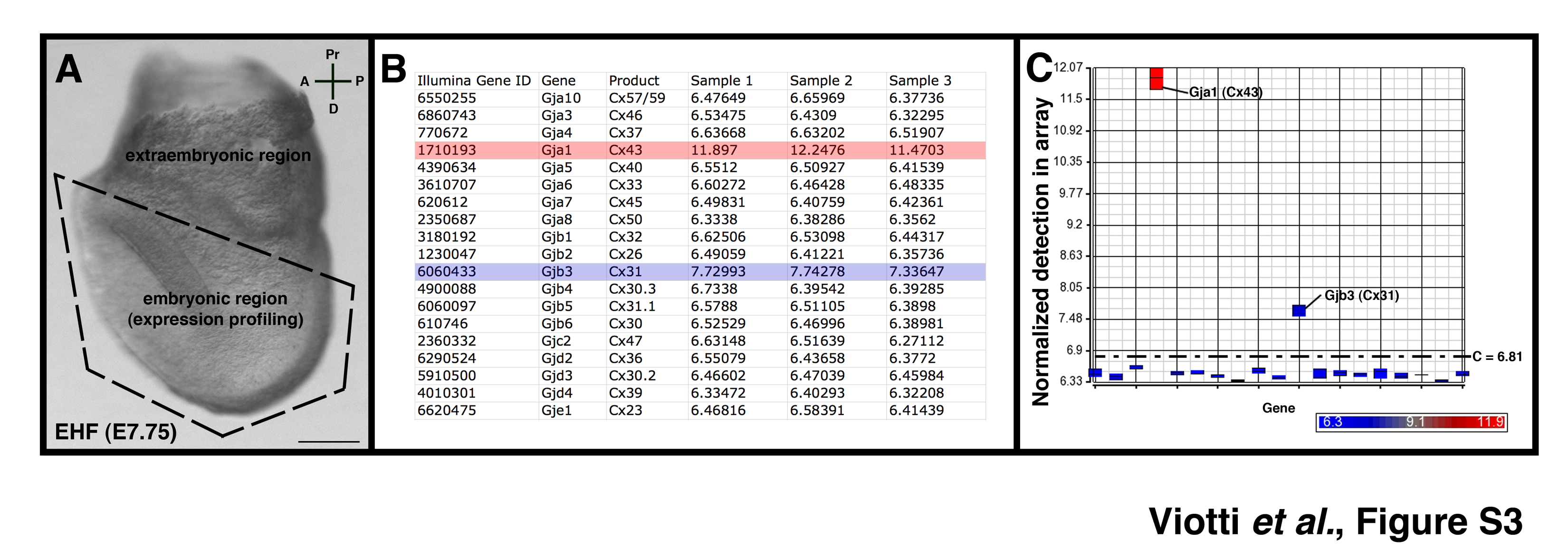

Supplement: Figure S3 — Gja1 (Cx43) is the predominant connexin expressed at the EHF stage. (A) Brightfield image of EHF (E7.75) embryo depicting region used for expression profiling. (B) Tabulated normalized detection levels of genes encoding connexins in expression array (p-value <0.01). (C) Graphical representation of connexins gene expression levels indicating high expression of Gja1 (Cx43) and low expression of Gjb3 (Cx31). Expression levels for all other connexin genes fell below background cut-off (dashed line, C = 6.81). A, anterior; D, distal; P, posterior; Pr, proximal. Scale bar = 100 µm. (TIF) [file pbio.1001276.s003.tif]

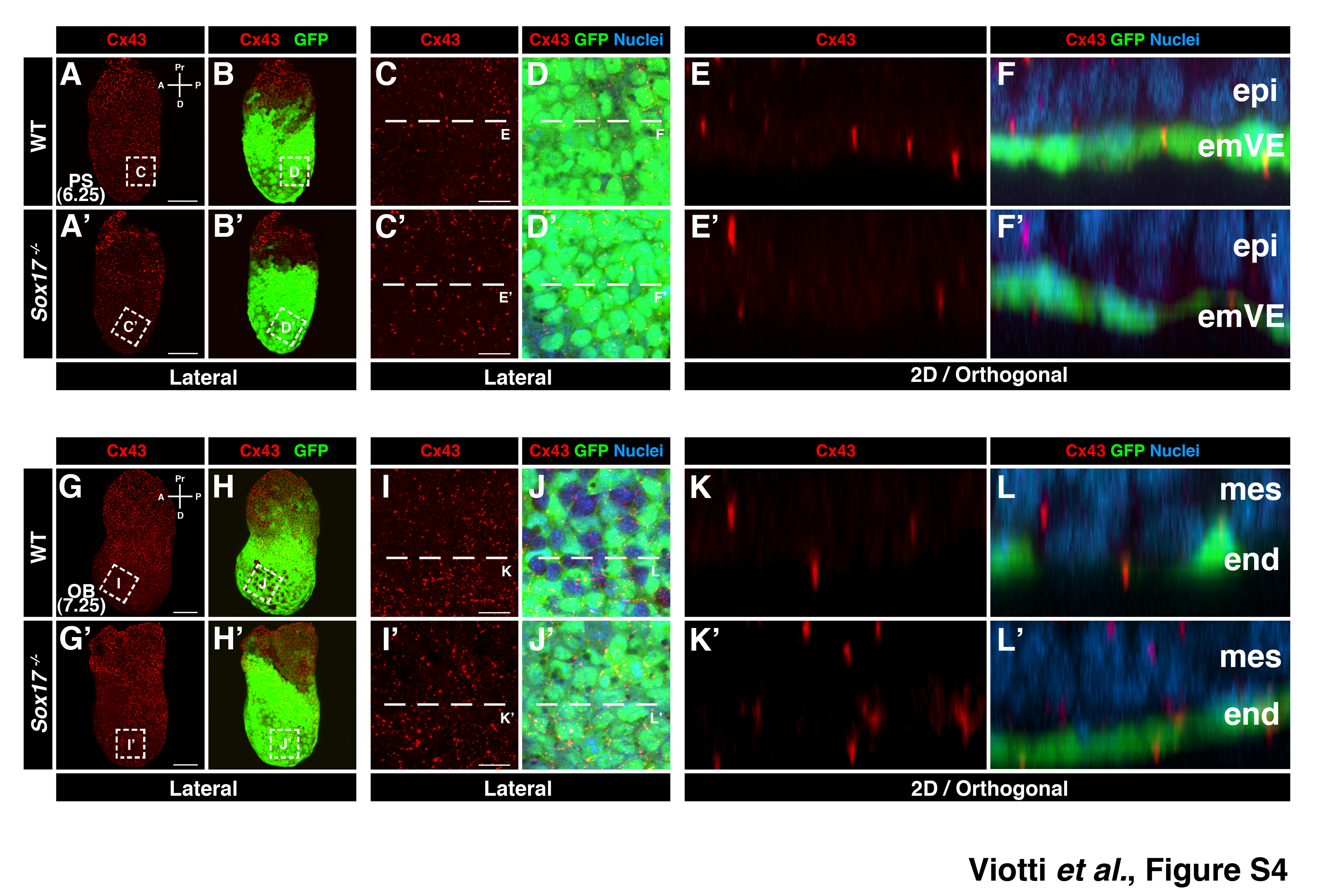

Supplement: Figure S4 — Cx43 localization in Sox17 mutants is normal until the onset of endoderm morphogenesis. (A and B) MIPs of confocal z-stacks of PS stage (6.25) wild-type and Sox17 mutant embryos immunofluorescently stained for the gap junction component Cx43. (C, C′, D, D′) High magnifications of the emVE region showing Cx43-positive puncta in the wild-type as well as the Sox17 mutant. (E, E′, F, F′) 2-D/orthogonal views of dashed lines in (C–D′) showing Cx43-positive puncta in the mesoderm and endoderm layers of the wild-type as well as the Sox17 mutant. (G and H) Lateral views of OB stage (E7.25) wild-type embryos stained for Cx43, showing first stages of emVE dispersal. (I and J) High magnifications of the dashed boxes in (G) and (H) showing Cx43-positive puncta in the endoderm layer, which is in the process of being dispersed. (K and L) 2-D/orthogonal views of the dashed lines in (I) and (J) showing Cx43-positive puncta in the mesoderm layer as well as between cells of the endoderm layer. (G′ and H′) Lateral views of an OB stage (E7.25) Sox17 mutant stained for Cx43. (I′ and J′) High magnifications of the dashed boxes in (G′) and (H′), showing CX43-positive puncta amongst undispersed emVE cells. (K′ and L′) 2-D/orthogonal views through the dashed line in (I′) and (J′) showing Cx43-positive puncta in the mesoderm layer as well as between undispersed emVE cells. emVE, embryonic VE; A, anterior; D, distal; P, posterior; Pr, proximal. Scale bars = 50 µm in (A, A′, G, and G′); 20 µm in (C, C′, I, and I′). (TIF) [file pbio.1001276.s004.tif]
