# Supplementary material for: Role of the Gut Endoderm in Relaying Left-Right Patterning in Mice
Source: PLoS Biol. 2012 Mar 6;10(3):e1001276. doi: 10.1371/journal.pbio.1001276 (PMC3295824; doi:10.1371/journal.pbio.1001276)
Supplement: Table S1 — Tabulation of experimental results. (DOC) [file pbio.1001276.s012.doc]

| Marker | Embryo | *n* | Gene expression | | |
| --- | --- | --- | --- | --- | --- |
|  |  |  | normal | reduced | absent |
| *Nodal* | Wild-type or *Sox17*^+/-^ | 14 | 14 | 0 | 0 |
|  | *Sox17*^-/-^ | 8 | 0 | 5 | 3 |
|  |  |  |  |  |  |
| *Lefty1/2* | Wild-type or *Sox17*^+/-^ | 15 | 14 | 1 | 0 |
|  | *Sox17*^-/-^ | 9 | 0 | 5 | 4 |
|  |  |  |  |  |  |
| *Pitx2* | Wild-type or *Sox17*^+/-^ | 7 | 7 | 0 | 0 |
|  | *Sox17*^-/-^ | 5 | 0 | 0 | 5 |
|  |  |  |  |  |  |
| *Nodal^LacZ/+^* | Wild-type or *Sox17*^+/-^ | 6 | 6 | 0 | 0 |
|  | *Sox17*^-/-^ | 4 | 0 | 0 | 4 |

| Marker | Treatment in culture | *n* | Gene expression | | | |
| --- | --- | --- | --- | --- | --- | --- |
|  |  |  | normal | reduced | absent | bilateral |
| *Lefty1/2* | No inhibitor | 14 | 11 | 2 | 0 | 1 |
| *Lefty1/2* | 18 alpha-Glycyrrhetinic acid  (General Gap Junction Inhibitor) | 14 | 1 | 3 | 10 | 0 |
| *Lefty1/2* | Mefloquine hydrochloride (Inhibitor specific to Cx36 and Cx50) | 11 | 8 | 1 | 1 | 1 |
